# The Association Between Presleep and Postwake Mobile Phone Use and Nonsuicidal Self-Injury Among University Students: Cross-Sectional Study

**DOI:** 10.2196/70819

**Published:** 2025-10-17

**Authors:** Xue Wang, Lei Zhang, Mingyang Wu, Xiaoxiao Yuan, Le Ma, Wenhua Wang

**Affiliations:** 1 School of General Medicine Xi'an Medical University Xi'an China; 2 Shaanxi Provincial Health Industry Association Service Center Xi'an China; 3 Shaanxi Medical Association Xi'an China; 4 Department of Maternal and Child Health Xiangya School of Public Health Central South University Changsha China; 5 School of Public Health, Xi’an Jiaotong University Health Science Center Xi'an China

**Keywords:** presleep mobile phone use, postwake mobile phone use, nonsuicidal self-injury, university students, dose-response association, cross-sectional study, sex differences, risk factor, sleep disruption, mental health

## Abstract

**Background:**

Nonsuicidal self-injury (NSSI) is a critical public health concern among university students, often considered a gateway behavior to suicide. With the widespread use of mobile phones, understanding the association between specific mobile phone use behaviors (eg, presleep and postwake mobile phone use) and NSSI has become increasingly important for targeted prevention.

**Objective:**

This study aimed to explore the association between presleep and postwake mobile phone use and NSSI among Chinese university students, examining potential dose-response relationships and sex differences.

**Methods:**

A multistage random cluster sampling survey was conducted across 6 universities in Shaanxi province (northwest China) from October 2022 to November 2022. A total of 18,585 undergraduates were included in the final analysis. Binary logistic regression models were used to examine the association between presleep and postwake mobile phone use duration and past-month NSSI, whereas restricted cubic spline regression was applied to assess dose-response relationships.

**Results:**

The prevalence of past-month NSSI among participants was 3.81% (709/18,585). Compared with individuals who reported lower presleep mobile phone use (0-30 minutes per day), those with higher presleep mobile phone use had substantially increased odds of NSSI, with odds ratios of 1.34 (95% CI 1.07-1.66) for the group with 61 to 120 minutes per day of use and 1.93 (95% CI 1.53-2.42) for the group with ≥120 minutes per day of use. For postwake mobile phone use, compared with the group with 0 to 1 minute per day of use, the participants in the group with >30 minutes per day of use showed a significant association with NSSI (odds ratio 1.27, 95% CI 1.02-1.58) in the fully adjusted model. Continuous variable analyses revealed that each 10-minute increase in presleep and postwake use was associated with a 3% and 2% higher NSSI risk, respectively. Restricted cubic spline analysis confirmed linear dose-response relationships for both presleep and postwake use (*P*>.05 for nonlinearity). No significant sex differences were observed in these associations.

**Conclusions:**

Prolonged presleep and postwake mobile phone use exhibited linear associations with NSSI among Chinese university students, with no significant sex disparities. These findings underscore the necessity of longitudinal studies to establish causality, elucidate underlying mechanisms, and inform targeted interventions.

## Introduction

### Background

Nonsuicidal self-injury (NSSI) is defined as the intentional, direct infliction of harm on one’s own body without suicidal intent, encompassing behaviors such as cutting, burning, scratching, hitting, and banging [1]. The prevalence of NSSI among adolescents worldwide varies between 7.5% and 46.5% [2,3], with a US survey of 61,767 university students reporting an annual incidence of 7.3% [4]. NSSI is widely recognized as a critical indicator of suicide risk in university students [5]. Those who engage in NSSI are 2.8 times more likely to experience suicidal thoughts and 5.5 times more likely to attempt suicide [6]. As such, NSSI constitutes a major global public health challenge [7], underscoring the need to identify its risk factors to develop effective prevention strategies.

Mobile phone use has become ubiquitous in modern society, with university students exhibiting particularly high dependency due to unique stressors, including academic pressure, social isolation, and identity formation [8]. Its impact on psychological and behavioral outcomes represents a global public health concern. Previous research has identified three mechanistic pathways: (1) neurobiological alterations, including dysregulation of the hypothalamic-pituitary-adrenal axis, reduced 5-hydroxytryptamine levels, impaired white matter integrity, and enhanced insula connectivity, all of which mediate maladaptive cognition and emotional dysregulation, leading to adverse psychological outcomes [9-11]; (2) circadian and affective disruption, manifested as biorhythm disturbances, insomnia, nightmares, poor sleep quality [12,13], emotional dysregulation, neglect, and rumination [14,15], all of which collectively contribute to anxiety, depression [16], NSSI, and suicide [14]; and (3) interpersonal modifications, including altered communication patterns, social comparison, social jet lag [17], diminished social support, and isolation [18], all of which are associated with adverse psychological sequelae [19]. Although the impact of mobile phone use on psychological and behavioral outcomes has received substantial attention, the relationship between mobile phone use during specific periods and NSSI remains inadequately explored. Previous studies have primarily examined the associations between NSSI and either total mobile phone use time or mobile phone addiction [20-29]. However, the interaction of person-affect-cognition-execution model [30] posits a progression from normative use to habitual behaviors to addiction. Specific behaviors (eg, presleep and postwake mobile phone use) serve as distinct indicators of use habits and key intermediate variables between normal use and addiction. Notably, current studies focusing on total mobile phone use time and mobile phone addiction have limitations in that they fail to account for the differential effects of distinct habitual behaviors and temporal variations in psychological impact, with equivalent use duration exerting divergent effects depending on whether it occurs during the day or night. For instance, presleep mobile phone use is demonstrably associated with bedtime procrastination, impaired sleep quality [31,32], nonrestorative sleep, and daytime tiredness [33]. Concurrently, postwake use is considered a behavioral withdrawal symptom stemming from nocturnal overuse [34-36], and this pattern may impair emotional regulation; exacerbate daily stress and psychological burden [37]; and correlate with morning fatigue, productivity loss, and psychological distress [38-40]. Collectively, presleep and postwake mobile phone use exert distinct adverse effects on mental health.

Several studies have explored the association between presleep and postwake mobile phone use and mental health outcomes. Presleep mobile phone use correlates with elevated anxiety and depression [31,38,41], whereas postwake mobile phone use exacerbates psychological distress [38]. A Hong Kong survey (N=3162) demonstrated that e-device use immediately after waking up and use for a long duration before sleeping were associated with anxiety and depressive symptoms, with combined exposure conferring the greatest risk [38]. Given NSSI’s ties to underlying psychological mechanisms, investigating these specific mobile phone use patterns and their association with NSSI is warranted. Both use windows are associated with insomnia and reduced sleep duration [42,43] through mechanisms such as melatonin suppression, circadian phase delay, and heightened cognitive arousal via blue light exposure [44-46], and poor sleep has been recognized as a critical predictor of NSSI [47]. Therefore, we hypothesized that presleep and postwake mobile phone use may be associated with an increased risk of NSSI. However, to date, only 1 study has explicitly examined the association between presleep mobile phone use and NSSI [48]. This was a 2008-2009 cross-sectional survey of Japanese adolescents that found that frequent post–lights-out mobile phone users had a 1.56-fold higher risk of NSSI. However, this work is limited by its measurement approach (categorizing frequency as “never,” “sometimes,” or “daily”), its sample population (consisting of adolescents rather than university students), and its historical context (a presmartphone era with divergent use patterns). Therefore, contemporary objective assessments are essential to further elucidate the association between these specific mobile phone use behaviors and NSSI.

### Objectives

This study investigated the associations between duration-based presleep and postwake mobile phone use and NSSI among Chinese university students, hypothesizing that prolonged mobile phone use during these specific periods confers NSSI vulnerability. Clarifying these behavioral pathways may yield targeted interventions to mitigate NSSI risk in this psychologically vulnerable population.

## Methods

### Design and Participants

This cross-sectional study used multistage random cluster sampling to recruit undergraduates from Shaanxi province, northwest China, from October 2022 to November 2022. Of the 57 provincial universities, 6 (11%) were randomly selected (n=4, 67% public; n=2, 33% private), proportionally representing institutional types. Within these universities, 559 classes were proportionally sampled across academic years, with all enrolled students invited. The initial recruitment yielded 18,723 participants from the sampled classes.

Six standardized training sessions were conducted for class representatives (2 per selected class), who subsequently guided their peers through survey completion. Data were collected via Wenjuanxing, a validated online survey platform, using unique access links. The research team developed the structured questionnaire using established scales, pretesting it with 300 nonsampled undergraduates to refine clarity. The final version included 13 domains with 196 items, taking approximately 27 minutes to complete. For the online survey, students could review the answered questions but not revise the responses; all questions were mandatory, with no skip logic.

**Figure 1:**
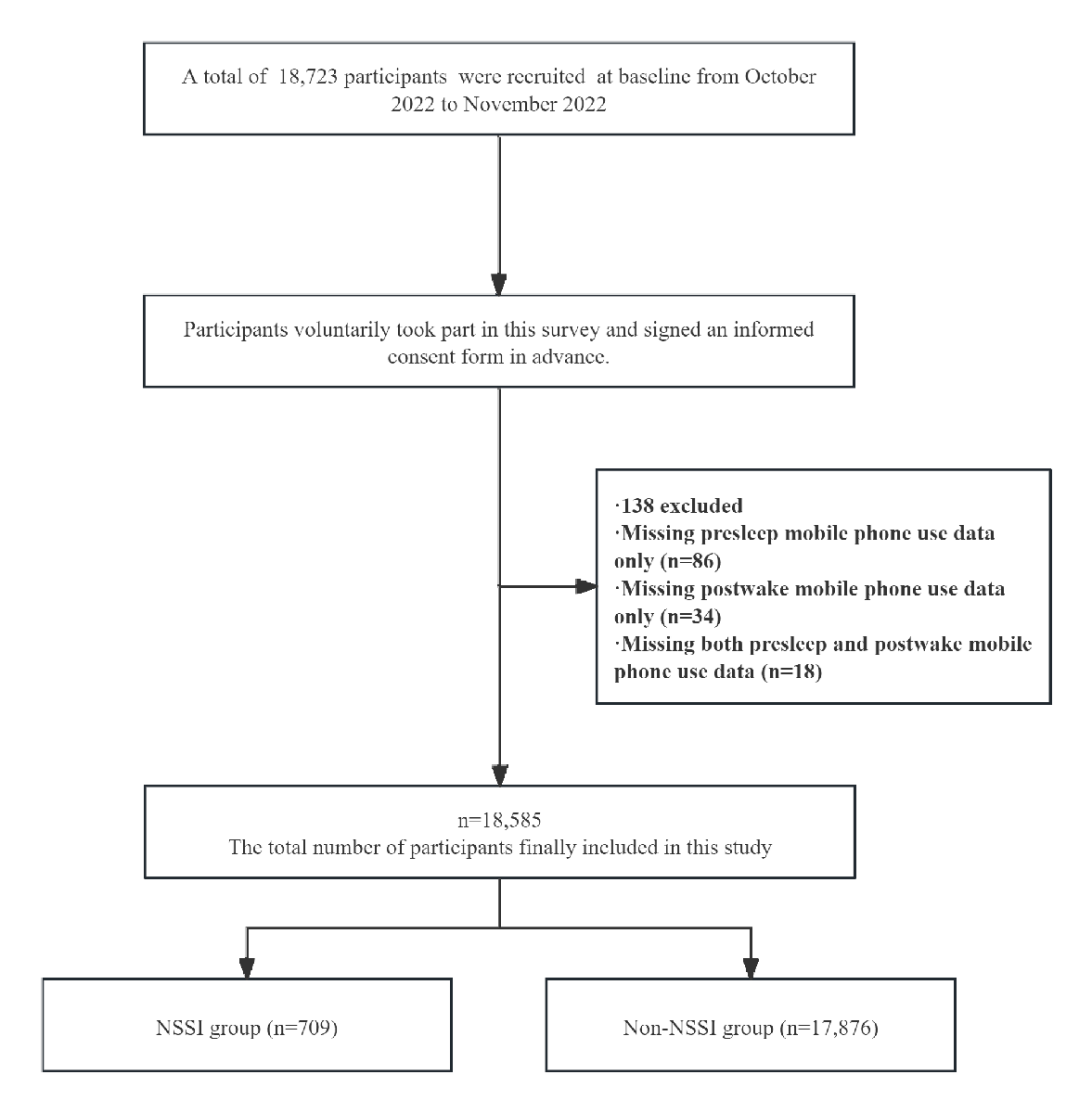
Flowchart. NNSI: nonsuicidal self-injury.

Eligibility criteria required participants to (1) be enrolled as undergraduates at the sampled universities, (2) belong to the selected classes, and (3) possess independent questionnaire completion capability. Exclusion criteria comprised survey completion refusal, incomplete submissions, implausibly rapid completion (<500 seconds, indicating inattention), logical inconsistencies, and missing key variable information. Following exclusions, the final analytic sample comprised 18,585 students. The participant flowchart is shown in Figure 1.

### Ethical Considerations

This study was approved by the ethics committee of the Second Affiliated Hospital of Xi’an Jiaotong University (approval 2022-248) and was conducted in accordance with the principles of the Declaration of Helsinki. Participants provided electronic informed consent after reviewing digital study materials, with verification audit trails. Data were anonymized after collection and secured in password-protected databases accessible only to authorized researchers. No financial incentives were provided.

### Questionnaire Content and Evaluation Method

#### Demographic Characteristics

Seven self-reported demographic variables were collected (categorical; reported as frequencies and percentages): sex (male or female), university year (first, second, third, or fourth and above), ethnicity (Han or other), registered permanent residence (rural or urban), siblings (yes or no), and parental educational attainment (mother and father; middle school or lower, high school, or college or higher).

#### Presleep and Postwake Mobile Phone Use Duration

Presleep mobile phone use was defined as the duration of mobile phone use while in bed before falling asleep, and postwake mobile phone use was defined as the duration of mobile phone use after awakening but before getting out of bed in the morning. Participants reported average daily durations over the preceding 7 days via a purpose-designed instrument, with responses recorded in minutes. This time frame balanced recall accuracy against typical use pattern capture [49], aligning with established protocols [16,50]. On the basis of data distribution, previous literature, and practical guidelines, presleep use was categorized into 0 to 30 minutes per day, 31 to 60 minutes per day, 61 to 120 minutes per day, and ≥120 minutes per day, whereas postwake use was classified into 0 to 1 minute per day, 2 to 10 minutes per day, 11 to 30 minutes per day, and >30 minutes per day.

#### NSSI Measurement

NSSI was measured using the Ottawa Self-Injury Inventory [51], focusing on the frequency of 10 specific behaviors, including hitting, biting, scratching, pulling out hair, cutting, burning, submerging or burying one's head in water to cause suffocation, tying oneself up with a rope, overusing or misusing drugs (not for treating illness), swallowing or drinking something inedible, in the previous month without suicidal intent. The Cronbach α coefficient of the Chinese version of the Ottawa Self-Injury Inventory is 0.952 [52]; in this study, the Cronbach α coefficient was 0.94.

#### Lifestyle Covariates

Lifestyle risk factors (smoking, drinking, unhealthy diet, and low level of physical activity) that are linked to NSSI risk [53-55] were assessed as covariates. Smoking and drinking were measured as binary variables (yes or no) based on self-reported behavior during the previous 30 days. Unhealthy diet was defined as failing to meet all 3 of the following criteria over a 2-week period [56,57]: red meat consumption <7 times per week, fresh fruit intake ≥7 times per week, and fresh vegetable intake ≥7 times per week. Physical activity levels were assessed using the validated International Physical Activity Questionnaire–Short Form [58], a tool widely applied in epidemiological research. Total physical activity was quantified as metabolic equivalent of task (MET) minutes per week by adding the time spent walking and engaging in moderate- and vigorous-intensity activities. Participants were stratified into 3 groups according to the International Physical Activity Questionnaire–Short Form standard protocols: a high activity level was defined as either ≥3 days per week of vigorous activity totaling ≥1500 MET minutes per day or ≥7 days per week of any combination of activities totaling ≥3000 MET minutes per week; a moderate activity level was defined as ≥3 days per week of vigorous activity (≥20 minutes per day), ≥5 days per week of moderate activity or walking (≥30 minutes per day), or ≥5 days per week of any combination of activities totaling ≥600 MET minutes per day; and a low physical activity level was defined as not meeting the criteria for moderate or high activity levels.

### Statistical Analysis

Descriptive statistics were used to characterize categorical variables as frequencies and percentages using the chi-square test for group comparisons. Continuous variables were subjected to normality and homogeneity tests. Normally distributed variables were expressed as means and SDs and compared using the 2-tailed *t* test or 1-way ANOVA, and nonparametric data were reported as medians and IQRs using the Wilcoxon rank sum test.

Binary logistic regression models were used to estimate the association between presleep and postwake mobile phone use and the likelihood of NSSI, with odds ratios (ORs) and 95% CIs reported. Analysis included both categorical and continuous forms of mobile phone use adjusted for potential covariates such as sex, university year, ethnicity, registered permanent residence, whether the participants had siblings, parental educational attainment, smoking status, drinking status, unhealthy diet, and low level of physical activity. The estimated coefficients associated with each 10-minute increase in exposure were presented, and trend tests were conducted by treating the categorized groups as ordinal variables in the logistic regression [59,60]. In addition, restricted cubic spline (RCS) regression was used to model dose-response association between presleep and postwake mobile phone use and NSSI. Moreover, acknowledging the sex-based disparities in the occurrence of NSSI and mobile phone use [28], subgroup analyses were conducted to examine sex-specific associations. We included the term sex × mobile phone use to obtain the *P* value for the interaction in the full adjustment models.

To mitigate potential bias from negative life events (eg, family misfortune, hospitalization, exam failure, and failed romantic relationships) in the previous year on the associations between mobile phone use and NSSI, we conducted a sensitivity analysis by incorporating these negative life events as covariates into the models. In addition, given the low frequency of NSSI occurrences over the previous month, we conducted a sensitivity analysis involving NSSI cases in the previous 6 and 12 months to analyze their relationship with mobile phone use. Statistical significance was set at a 2-sided *P* value of <.05. Statistical analyses were conducted using R (version 4.4.2; R Foundation for Statistical Computing).

## Results

### Participant Characteristics

A total of 18,585 university students were included, with 6476 (34.84%) being male and 12,109 (65.15%) being female. First-, second-, third-, and fourth-year and above students accounted for 29.13% (5414/18,585), 23.81% (4426/18,585), 23.5% (4369/18,585), and 23.54% (4376/18,585), respectively. Median presleep mobile phone use duration was significantly higher among students with experience of NSSI (90, IQR 60-137 minutes) than among those without (60, IQR 30-120 minutes). Similarly, postwake use duration was elevated in the NSSI group (20, IQR 5-60 minutes) compared to the non-NSSI group (15, IQR 2-45 minutes). The 1-month prevalence rate of NSSI was 3.81% (709/18,585), with no significant sex differences. The comparative characteristics of participants with and without experience of NSSI are shown in Table 1, with sex-stratified analyses provided in Multimedia Appendix 1.

**Table 1:** Characteristics of the participants with and without experience of nonsuicidal self-injury (NSSI)^a^.

Characteristic				NSSI group (n=709)		Non-NSSI group (n=17,876)		*P* value	
**Participant characteristics, n (%)**									
	**Sex**								.57
		Male	240 (33.9)		6236 (34.9)				
		Female	469 (66.1)		11,640 (65.1)				
	**University year**								.02
		First	164 (23.1)		5250 (29.4)				
		Second	198 (27.9)		4228 (23.7)				
		Third	176 (24.8)		4193 (23.5)				
		Fourth and above	171 (24.1)		4205 (23.5)				
	**Ethnicity**								.36
		Han	684 (96.5)		17,351 (97.1)				
		Others	25 (3.5)		525 (2.9)				
	**Registered permanent residence**								<.001
		Rural	339 (47.8)		9705 (54.3)				
		Urban	370 (52.2)		8171 (45.7)				
**Family characteristics, n (%)**									
	**Siblings**								.03
		Yes	235 (33.1)		5252 (29.4)				
		No	474 (66.9)		12,624 (70.6)				
	**Maternal educational attainment**								.26
		Middle school or lower	438 (61.8)		11,520 (64.4)				
		High school	148 (20.9)		3614 (20.2)				
		College or higher	123 (17.3)		2742 (15.3)				
	**Paternal educational attainment**								.28
		Middle school or lower	371 (52.3)		9799 (54.8)				
		High school	173 (24.4)		3936 (22.0)				
		College or higher	165 (23.3)		4141 (23.2)				
**Unhealthy lifestyle characteristics, n (%)**									
	Smoking			140 (19.7)		2094 (11.7)		<.001	
	Drinking			266 (37.5)		3272 (18.3)		<.001	
	Unhealthy diet			659 (92.9)		16,511 (92.4)		.57	
	Low level of physical activity			538 (75.9)		13,249 (74.1)		.29	
**Mobile phone use**									
	Presleep mobile phone use (minutes), median (IQR)			90 (60-137)		60 (30-120)		<.001	
	**Presleep mobile phone use (minutes per day), n (%)**								<.001
		0-30	135 (19)		4789 (26.8)				
		31-60	144 (20.3)		4349 (24.3)				
		61-120	228 (32.2)		5534 (31.0)				
		>120	202 (28.5)		3204 (17.9)				
	Postwake mobile phone use (minutes), median (IQR)			20 (5-60)		15 (2-45)		<.001	
	**Postwake mobile phone use (minutes per day), n (%)**								.01
		0-1	147 (20.7)		4414 (24.7)				
		2-10	161 (22.7)		4109 (23.0)				
		11-30	179 (25.2)		4675 (26.2)				
		>30	222 (31.3)		4678 (26.2)				

^a^For the statistical analysis of the features in this table, *t* tests, ANOVA, or Wilcoxon tests were used for continuous variables, and chi-square tests were used for categorical variables. A *P* value of <.05 indicates statistical significance.

### Associations Between Presleep and Postwake Mobile Phone Use and NSSI

Both presleep and postwake mobile phone use demonstrated significant dose-dependent associations with NSSI risk. Compared to the reference group (0-30 minutes per day), higher presleep mobile phone use categories showed elevated NSSI risk across all models. In the fully adjusted model 3, those with higher presleep mobile phone use had significantly increased odds of NSSI, with ORs of 1.34 (95% CI 1.07-1.66) for the group with 61 to 120 minutes per day of use and 1.93 (95% CI 1.53-2.42) for the group with ≥120 minutes per day of use (*P*<.001 for the trend). Each 10-minute daily increase in presleep use conferred 3% higher NSSI risk (OR 1.03, 95% CI 1.02-1.04).

For postwake mobile phone use, relative to the reference group (0-1 minute per day), the highest-exposure group (>30 minutes per day) showed significantly elevated risk in fully adjusted model 3 (OR 1.27, 95% CI 1.02-1.58), whereas intermediate categories (2-10 minutes per day: OR 1.11, 95% CI 0.88-1.40; 11-30 minutes per day: OR 1.06, 95% CI 0.85-1.33) were nonsignificant. Each 10-minute increment in postwake use was associated with a 2% higher risk (OR 1.02, 95% CI 1.01-1.03). Detailed results are shown in Table 2.

**Figure 2:**
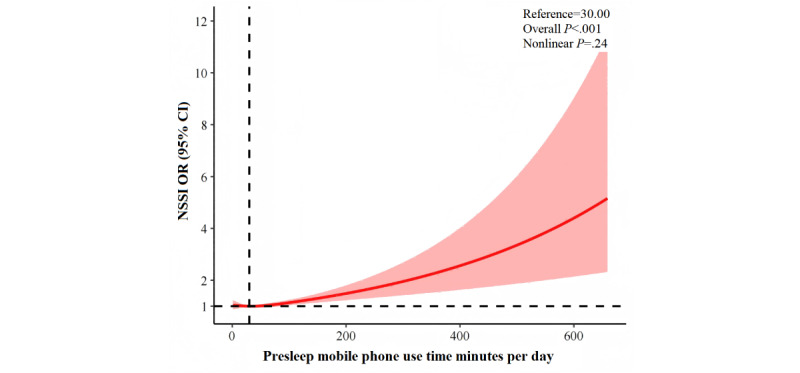
Restricted cubic splines regression analysis of presleep and mobile phone use with NSSI. NSSI: nonsuicidal self-injury; OR: odds ratio.

**Figure 3:**
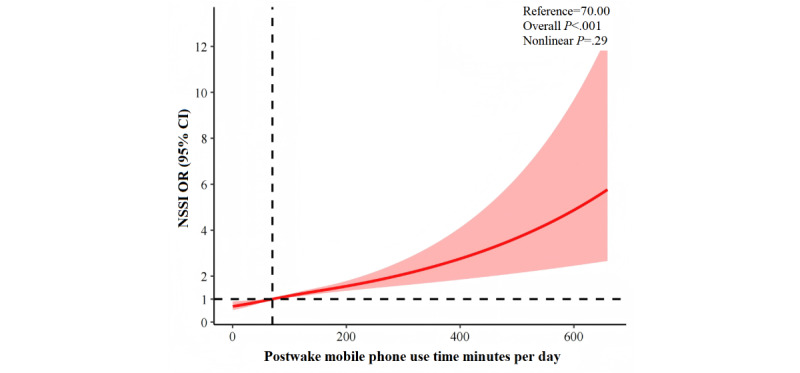
Restricted cubic splines regression analysis of postwake mobile phone use with NSSI. NSSI: nonsuicidal self-injury; OR: odds ratio.

RCS models confirmed linear relationships for both presleep (nonlinear *P*=.24; Figure 2) and postwake (nonlinear *P*=.30; Figure 3) use, with risk increasing steadily with use duration.

**Table 2:** Associations between presleep and postwake mobile phone use and past–1-month nonsuicidal self-injury (NSSI).

		Participants per group, n	NSSI prevalence, n (%)	Model 1^a^, OR^b^ (95% CI)	Model 2^c^, OR (95% CI)	Model 3^d^, OR (95% CI)
**Presleep mobile phone use time (minutes per day)^e^**						
	0-30	4924	135 (2.74)	Reference	Reference	Reference
	31-60	4493	144 (3.2)	1.18 (0.93-1.49)	1.16 (0.92-1.48)	1.10 (0.86-1.39)
	61-120	5762	228 (3.95)	1.46 (1.18-1.82)	1.48 (1.19-1.83)	1.34 (1.07-1.66)
	>120	3406	202 (5.93)	2.24 (1.79-2.79)	2.28 (1.83-2.86)	1.93 (1.53-2.42)
Presleep mobile phone use time (increase by 10 min per d)		—^f^	—	1.04 (1.03-1.05)	1.04 (1.03-1.05)	1.03 (1.02-1.04)
**Postwake mobile phone use time (minutes per day)^g^**						
	0-1	4561	147 (3.22)	Reference	Reference	Reference
	2-10	4270	161 (3.77)	1.18 (0.94-1.48)	1.18 (0.94-1.48)	1.11 (0.88-1.40)
	11-30	4854	179 (3.68)	1.15 (0.92-1.44)	1.17 (0.94-1.47)	1.06 (0.85-1.33)
	＞30	4900	222 (4.53)	1.43 (1.15-1.76)	1.46 (1.18-1.81)	1.27 (1.02-1.58)
Postwake mobile phone use time (increase by 10 minutes per day)		—	—	1.03 (1.02-1.04)	1.03 (1.02-1.04)	1.02 (1.01-1.03)

^a^Unadjusted.

^b^OR: odds ratio.

^c^Adjusted for sex, university year, ethnicity, registered permanent residence, having siblings, maternal educational attainment, and paternal educational attainment.

^d^Adjusted for sex, university year, ethnicity, registered permanent residence, having siblings, maternal educational attainment, paternal educational attainment, smoking status, drinking status, unhealthy diet, and low level of physical activity.

^e^*P*<.001 for models 1 to 3 for the trend.

^f^Not applicable.

^g^*P*<.001 for model 1, *P*<.001 for model 2, and *P*=.02 for model 3 for the trend.

### Sex-Stratified Analyses

Sex-stratified analyses revealed differential associations (Table 3). For presleep mobile phone use, the interaction term was nonsignificant (*P*=.45 for the interaction), confirming consistent associations across sexes. For postwake use, while the interaction term approached significance (*P*=.46 for the interaction), stratified results showed no significant associations with NSSI in either sex.

**Table 3:** Associations between presleep and postwake mobile phone use and nonsuicidal self-injury stratified by sex^a^.

		Male individuals, OR^b^ (95% CI)		Female individuals, OR (95% CI)	*P* value for the interaction
**Presleep mobile phone use (minutes per day)^c^**					
	0-30	Reference	Reference		—^d^
	31-60	1.16 (0.79-1.72)	1.05 (0.78-1.43)		—
	61-120	1.47 (1.02-2.13)	1.25 (0.95-1.65)		—
	>120	2.11 (1.42-3.14)	1.80 (1.36-2.38)		—
Presleep mobile phone use (increase by 10 minutes per day)		1.03 (1.02-1.05)		1.03 (1.02-1.05)	.45
**Postwake mobile phone use time (minutes per day)^e^**					
	0-1	Reference	Reference		—
	2-10	1.28 (0.87-1.90)	1.04 (0.78-1.38)		—
	11-30	1.19 (0.82-1.73)	1.01 (0.76-1.34)		—
	＞30	1.37 (0.95-1.98)	1.21 (0.92-1.58)		—
Postwake mobile phone use (increase by 10 minutes per day)		1.02 (1.00-1.04)		1.02 (1.01-1.04)	.46

^a^Adjusted for university year, ethnicity, registered permanent residence, having siblings, maternal educational attainment, paternal educational attainment, smoking status, drinking status, unhealthy diet, and low level of physical activity.

^b^OR: odds ratio.

^c^*P*<.001 for male and female individuals for the trend.

^d^Not applicable.

^e^*P*=.14 for male individuals and *P*=.18 for female individuals for the trend.

### Sensitivity Analyses

Sensitivity analyses confirmed the robustness of our core findings. First, incorporating negative life events as covariates in the models did not materially alter the observed association between presleep mobile phone use and NSSI, reinforcing the stability of this primary result. However, for postwake mobile phone use, the association with NSSI was nonsignificant when analyzed as a categorical variable, whereas significance was retained in the continuous variable analysis (Multimedia Appendices 2 and 3). Second, supplementary analyses using 6- and 12-month NSSI recall periods yielded patterns consistent with the 1-month data, further validating the link between time-specific mobile phone use and NSSI (Multimedia Appendices 4 and 5).

## Discussion

### Principal Findings

This large-scale cross-sectional study examined the association between presleep and postwake mobile phone use and NSSI among Chinese university students. Key findings included the following: (1) prolonged presleep and postwake mobile phone use were significantly associated with NSSI (eg, ≥120 minutes of presleep use correlated with 93% higher odds of NSSI, and >30 minutes of postwake use correlated with 27% higher odds of NSSI), (2) RCS analyses confirmed a linear dose-response relationship between presleep and postwake mobile phone use and NSSI, and (3) there were no significant sex disparities in these associations.

### Comparison With Other Studies

Our sample exhibited a 1-month NSSI prevalence of 3.81% (709/18,585) among the participants, with 5.33% (991/18,585) of the participants having a 6-month prevalence and 6.58% (1223/18,585) of the participants having a 12-month prevalence (Multimedia Appendix 2). These rates align with global ranges (7.5%-46.5% in adolescents and 4%-23% in adults) [2]. University-specific comparisons further contextualize these findings: a US cohort survey of 61,767 university students reported a 7.3% annual NSSI incidence [4]. Another survey reported an 8% 12-month prevalence among female university students (n=1158) and a 2.2% 9-month incidence of new cases. Canadian data similarly indicated a 4.1% 12-month incidence (n=666) [47]. Notably, no previous studies have specified 1-month NSSI rates. Consistent with previous research showing that NSSI prevalence decreases with age, the NSSI prevalence in our study was lower than the 17.1% reported in a study of Chinese individuals aged 11 to 20 years (n=15,623) [23]. Our observed mobile phone use patterns align with established evidence of high nighttime engagement among youth [61,62]. Among university students, 1707 out of 1925 (88.67%) reported using their mobile phones after lights-out [63], and 715 out of 3162 (22.61%) reported using mobile phones within 5 minutes of waking [38]. Critically, our study advances beyond previous research on total mobile phone use duration or addiction as correlates of NSSI [20,22,25,64] by establishing temporally specific risk windows. Our presleep mobile phone use association substantiates the seminal finding by Oshima et al [48] of a 1.75-fold increase in NSSI risk among adolescents who used their phones after lights-out while providing a novel quantification that participants who used their phones for more than 2 hours before sleep had 93% higher odds of engaging in NSSI compared to those with less than 30 minutes of presleep phone use. Sensitivity analyses further revealed differential stability between use periods: while presleep use associations remained robust, postwake categorical associations trended toward nonsignificance after covariate adjustment, although continuous exposure trends persisted. This indicates that presleep use may constitute a more reliable behavioral marker for NSSI risk.

### Potential Mechanisms

Presleep and postwake mobile phone use may be associated with NSSI through 4 distinct yet interconnected mechanistic pathways. The first is the physiological pathway: sleep and circadian disruption initiates via blue light–mediated melatonin suppression that directly delays sleep onset [44,46]. Consequently, prolonged nighttime mobile phone use induces sleep compression, reducing total sleep duration [43], whereas subsequent notification-driven sleep fragmentation degrades restorative sleep quality [65]. Sleep impairment increases NSSI risk 3.24-fold [66], with presleep mobile phone use demonstrating a stronger association due to its direct interference with sleep initiation physiology. In contrast, postwake use exerts comparatively weaker effects through secondary circadian misalignment. In the second mechanistic pathway, neurobiological adaptations emerge through chronic presleep and postwake mobile phone exposure. Heightened dopaminergic signaling in reward pathways amplifies incentive salience, whereas reduced endogenous opioid availability blunts natural reward processing [9,67]. Concurrently, attenuated 5-hydroxytryptamine synthesis compromises mood regulation, and altered functional connectivity in the prefrontal-amygdala-insula circuits impairs emotional awareness [11]. Cumulatively, these neural alterations establish a vulnerability triad (blunted natural reward sensitivity, intensified digital craving, and lowered thresholds for maladaptive coping), ultimately facilitating NSSI as an alternative distress regulation strategy [9,67]. Third, psychological dysregulation manifests through attentional capture via digital content that prevents adaptive emotion processing [68,69], compounded by displacement of self-reflection through reactive scrolling that impedes cognitive reappraisal [70]. When sustained, this pattern reinforces avoidance-based coping strategies [68,69], establishing a self-perpetuating cycle as digital distraction fails to provide emotional relief, and escalating distress progressively increases NSSI as a termination mechanism [17]. Consequently, clinically observable deficits emerge, including intensified negative effect, prolonged emotional recovery latency, and diminished distress tolerance [71]. Fourth, behavioral contagion mechanisms operate distinctly through nighttime mobile phone use ecosystems, and algorithm-driven exposure to normalization narratives lowers behavioral inhibitions [72], whereas peer reinforcement in online communities validates maladaptive coping [70]. Furthermore, curated content consumption cognitively reframes NSSI as identity affirming [71]. Social contagion theory posits that these dynamics model behavior, provide vicarious reinforcement, and dismantle psychological barriers, effectively constructing digital microenvironments where NSSI acquires perceived social validation [71,72]. Critically, pathway activation demonstrates temporal specificity, and presleep mobile phone use primarily engages physiological and neurobiological pathways through direct interference with sleep initiation and neurochemical rhythms, whereas postwake mobile phone use predominantly activates psychological and behavioral pathways via immediate exposure to emotionally salient content upon awakening. This mechanistic segregation explains the differential effect magnitudes observed between the use periods. Future longitudinal investigations should quantify pathway-specific mediation effects to inform chronologically optimized interventions targeting these discrete vulnerability pathways.

### Sex Considerations

Despite female individuals reporting significantly longer presleep mobile phone use (*P*<.001), no sex-based disparities were observed in either NSSI prevalence or its association with presleep and postwake mobile phone use. This finding aligns with Asian epidemiological studies reporting comparable NSSI rates across sexes but contrasts with Western literature documenting elevated female NSSI incidence [3]. Notably, a study found sex-specific sleep-related NSSI risks in adolescents [73], but our university student sample showed no such pattern, potentially reflecting age-related changes in coping mechanisms.

### Limitations and Future Research

This study has several limitations. First, self-report measures may introduce recall bias, although objective tracking (eg, app-based data) could validate the findings [43]. Second, the cross-sectional design precludes causal inference, and reverse pathways cannot be ruled out. Third, uneven sex distribution may limit generalizability. Fourth, the selected time windows for mobile phone use and NSSI may limit the capture of long-term associations. Fifth, participant recruitment was conducted exclusively in China. Cultural context may limit the generalizability of the findings to university students in other countries. Sixth, despite adjustments for potential confounders in the models, residual confounding may persist due to unmeasured variables influencing both mobile phone use duration and NSSI risk. Future research should (1) adopt longitudinal designs with extended time windows to clarify causality, disentangle pathways, and capture long-term associations; (2) include unmeasured variables to mitigate confounding and explore mediators, such as sleep quality, emotion regulation neural circuitry, and content exposure; (3) include sex-balanced and culturally diverse samples from varied geographic regions to enhance generalizability across groups and contexts; and (4) conduct randomized interventions to evaluate protocols in university student cohorts.

### Conclusions

This cross-sectional study demonstrates that prolonged presleep mobile phone use (>60 minutes per day) and postwake mobile phone use (>30 minutes per day) exhibit significant associations with NSSI, with the latter showing slightly weaker stability in categorical analyses. No significant sex differences were observed. Consequently, prolonged presleep and postwake mobile phone use represent modifiable risk factors, necessitating longitudinal studies to establish causality, elucidate underlying mechanisms, and inform targeted interventions.

## Data Availability

The datasets generated or analyzed during this study are available from the corresponding author on reasonable request.

## Appendix

Multimedia Appendix 1 Sex-stratified characteristics of participants.

Multimedia Appendix 2 Characteristics of negative life events stratified by sex and nonsuicidal self-injury.

Multimedia Appendix 3 Sensitivity analysis including negative life events.

Multimedia Appendix 4 Sensitivity analysis of presleep and postwake mobile phone use and 6-month nonsuicidal self-injury.

Multimedia Appendix 5 Sensitivity analysis of presleep and postwake mobile phone use and 12-month nonsuicidal self-injury.

## Abbreviations

MET: metabolic equivalent of task
NSSI: nonsuicidal self-injury
OR: odds ratio
RCS: restricted cubic spline
