# Supplementary material for: The Association Between Presleep and Postwake Mobile Phone Use and Nonsuicidal Self-Injury Among University Students: Cross-Sectional Study
Source: J Med Internet Res. 2025 Oct 17;27:e70819. doi: 10.2196/70819 (PMC12579296; doi:10.2196/70819)
Supplement: Multimedia Appendix 1 [file jmir_v27i1e70819_app1.docx]

**Multimedia Appendix 1**: Sex-stratified characteristics of participants

| **Characteristics** | ***Male***  ***(n=6,476)*** | ***Female (n=12,109)*** | ***P*** |
| --- | --- | --- | --- |
| **Self-Characteristics** |  |  |  |
| Grade, n (%) |  |  | .45 |
| 1st | 1909 (29.5) | 3505 (28.9) |  |
| 2nd | 1535 (23.7) | 2891 (23.9) |  |
| 3rd | 1547 (23.9) | 2822 (23.3) |  |
| 4th+ | 1485 (22.9) | 2891 (23.9) |  |
| Ethnicity, n (%) |  |  | .22 |
| Han | 6298 (97.3) | 11737 (96.9) |  |
| others | 178 (2.7) | 372 (3.1) |  |
| Registered permanent residence, n (%) |  |  | .58 |
| rural | 3518 (54.3) | 6526 (53.9) |  |
| urban | 2958 (45.7) | 5583 (46.1) |  |
| **Family Characteristics** |  |  |  |
| Sibling, n (%) |  |  | **<.001** |
| Yes | 2343 (36.2) | 3144 (26.0) |  |
| No | 4133 (63.8) | 8965 (74.0) |  |
| Maternal educational attainment, n (%) |  |  | **.02** |
| middle school or under | 4090 (63.2) | 7868 (65.0) |  |
| high school | 1332 (20.6) | 2430(20.1) |  |
| college or above | 1054(16.3) | 1811 (15.0) |  |
| Paternal educational attainment, n (%) |  |  | **<.001** |
| middle school or under | 3377 (52.1) | 6793 (56.1) |  |
| high school | 1483 (22.9) | 2626 (21.7) |  |
| college or above | 1616 (25.0) | 2690 (22.2) |  |
| **Unhealthy Life style** |  |  |  |
| Smoking, n (%) | 1604 (24.8) | 630 (5.2) | **<.001** |
| Drinking, n (%) | 1814 (28) | 1724 (14.2) | **<.001** |
| Unhealthy Diet,n(%) | 6141 (94.8) | 11029 (91.1) | **<.001** |
| Less Physical Activity,n(%) | 4450 (68.7) | 9337 (77.1) | **<.001** |
| **Mobile Phone Use** |  |  |  |
| presleep mobile phone use, median (IQR) | 60(30,120) | 65(40,120) | **<.001** |
| presleep mobile phone use (minutes per day) |  |  | **<.001** |
| 0-30 | 1998(30.9) | 2926(24.2) |  |
| 31-60 | 1701(26.3) | 2792(23.1) |  |
| 61-120 | 1807(27.9) | 3955(32.7) |  |
| >120 | 970(15.0) | 2436(20.1) |  |
| postwake mobile phone use, median (IQR) | 10(0,30) | 20(3,60) | **.02** |
| postwake mobile phone use (minutes per day) |  |  | **<.001** |
| 0-1 | 1886(29.1) | 2675(22.1) |  |
| 2-10 | 1356(20.9) | 2914(24.1) |  |
| 11-30 | 1635(25.2) | 3219(26.6) |  |
| ＞30 | 1599(24.7) | 3301(27.3) |  |
| **NSSI** |  |  |  |
| Past 1-month NSSI, n(%) | 240 (3.7) | 469 (3.9) | .57 |
| Past 6-month NSSI, n(%) | 328 (5.0) | 663 (5.4) | .24 |
| Past 12-month NSSI, n(%) | 388 (5.9) | 835 (6.8) | **.02** |

^a^Use 2-tailed t tests, ANOVA or Wilcoxon tests for continuous variables, chi-square tests for categorical variables for statistical analysis of the features in the table. A *P-*value <.05 indicates statistical significance.
