# Supplementary material for: The Association Between Presleep and Postwake Mobile Phone Use and Nonsuicidal Self-Injury Among University Students: Cross-Sectional Study
Source: J Med Internet Res. 2025 Oct 17;27:e70819. doi: 10.2196/70819 (PMC12579296; doi:10.2196/70819)
Supplement: Multimedia Appendix 2 [file jmir_v27i1e70819_app2.docx]

**Multimedia Appendix 2**:

Characteristics of negative life events stratified by sex and NSSI status

| **Characteristics** | ***Male***  ***(n=6,476)*** | ***Female (n=12,109)*** | ***P*** | ***NSSI***  ***(n=709)*** | ***Non-NSSI (n=17,876)*** | ***P*** |
| --- | --- | --- | --- | --- | --- | --- |
| Family misfortune | 637 (9.8) | 1192 (9.8) | .99 | 112(15.8) | 1717(9.6) | **<.001** |
| Hospitalization | 581 (9.0) | 1017 (8.4) | .18 | 135(19.0) | 1463(8.2) | **<.001** |
| Exam failure | 3199 (49.4) | 5893 (48.7) | .34 | 451(63.6) | 8641(48.3) | **<.001** |
| Failed romantic relationships | 1436 (16.6) | 2008 (16.6) | **<.001** | 249(35.1) | 3195(17.9) | **<.001** |

^a^Use chi-square tests for categorical variables for statistical analysis of the features in the table. A *P-*value <.05 indicates statistical significance.
