# Supplementary material for: The Association Between Presleep and Postwake Mobile Phone Use and Nonsuicidal Self-Injury Among University Students: Cross-Sectional Study
Source: J Med Internet Res. 2025 Oct 17;27:e70819. doi: 10.2196/70819 (PMC12579296; doi:10.2196/70819)
Supplement: Multimedia Appendix 3 [file jmir_v27i1e70819_app3.docx]

**Multimedia Appendix 3:** Sensitivity analysis including negative life events

| Mobile phone use duration | *Model 4* |
| --- | --- |
|  | *OR（95% CI）* |
| Presleep mobile phone use time(minutes/day) |  |
| 0-30 | reference |
| 31-60 | 1.09（0.85-1.38） |
| 61-120 | **1.28（1.03-1.60）** |
| >120 | **1.74（1.38-2.20）** |
| *P_trend_* | <.001 |
| Presleep mobile phone use time(increase by 10 minutes per day) | **1.03（1.02-1.04）** |
| Postwake mobile phone use time(minutes per day) |  |
| 0-1 | reference |
| 2-10 | 1.08（0.86-1.36） |
| 11-30 | 1.03（0.82-1.30） |
| ＞30 | 1.17（0.94-1.46） |
| *P_trend_* | .11 |
| Postwake mobile phone use time(increase by 10 minutes per day) | **1.02（1.01-1.03）** |

^a^model 4: adjustment for sex, grade, ethnicity, registered permanent residence, sibship, maternal educational attainment, paternal educational attainment, smoking, drinking, unhealthy diet, less physical activity, family misfortune, hospitalization, exam failure, and failed romantic relationships.
