# Supplementary material for: The Association Between Presleep and Postwake Mobile Phone Use and Nonsuicidal Self-Injury Among University Students: Cross-Sectional Study
Source: J Med Internet Res. 2025 Oct 17;27:e70819. doi: 10.2196/70819 (PMC12579296; doi:10.2196/70819)
Supplement: Multimedia Appendix 4 [file jmir_v27i1e70819_app4.docx]

**Multimedia Appendix 4.** Sensitivity analysis of presleep and postwake mobile phone use and 6-month NSSI

| Mobile phone use duration | *model 1* | *model 2* | *model 3* |
| --- | --- | --- | --- |
|  | *OR（95% CI）* | *OR（95% CI）* | *OR（95% CI）* |
| Presleep mobile phone use time（minutes per day） |  |  |  |
| 0-30 | reference | reference | reference |
| 31-60 | 1.16  (0.95-1.42) | 1.57  (0.95-1.42) | 1.08  (0.88-1.33) |
| 61-120 | 1.45  (1.21-1.74) | 1.46  (1.21-1.75) | 1.31  (1.09-1.58) |
| >120 | 2.15  (1.78-2.60) | 2.19  (1.81-2.65) | 1.83  (1.50-2.23) |
| *P_trend_* | <.001 | <.001 | <.001 |
| Presleep mobile phone use time(increase by 10 minutes per day) | 1.04  (1.03-1.05) | 1.04  (1.03-1.05) | 1.03  (1.03-1.05) |
| Post-wake mobile phone use time(minutes per day) |  |  |  |
| 0-1 | reference | reference | reference |
| 2-10 | 1.25  (1.03-1.52) | 1.23  (1.02-1.51) | 1.17  (0.96-1.43) |
| 11-30 | 1.16  (0.95-1.40) | 1.16  (0.96-1.41) | 1.06  (0.87-1.28) |
| ＞30 | 1.50  (1.25-1.81) | 1.51  (1.26-1.82) | 1.32  (1.10-1.59) |
| *P_trend_* | <.001 | <.001 | .01 |
| Postwake mobile phone use time(increase by 10 minutes per day) | 1.03  (1.02-1.04) | 1.03  (1.02-1.04) | 1.02  (1.01-1.03) |

^a^model 1: unadjusted;

^b^model 2: adjustment for sex, grade, ethnicity, registered permanent residence, sibship, maternal educational attainment, paternal educational attainment;

^c^model 3: adjustment for sex, grade, ethnicity, registered permanent residence, sibship, maternal educational attainment, paternal educational attainment, smoking, drinking, unhealthy diet, and less physical activity.
