# Supplementary material for: The Association Between Presleep and Postwake Mobile Phone Use and Nonsuicidal Self-Injury Among University Students: Cross-Sectional Study
Source: J Med Internet Res. 2025 Oct 17;27:e70819. doi: 10.2196/70819 (PMC12579296; doi:10.2196/70819)
Supplement: Multimedia Appendix 5 [file jmir_v27i1e70819_app5.docx]

**Multimedia Appendix 5.** Sensitivity analysis of presleep and postwake mobile phone use and 12-month NSSI

| Mobile phone use duration | *model 1* | *model 2* | *model 3* |
| --- | --- | --- | --- |
|  | *OR（95% CI）* | *OR（95% CI）* | *OR（95% CI）* |
| Presleep mobile phone use time(minutes per day) |  |  |  |
| 0-30 | reference | reference | reference |
| 31-60 | 1.22  (1.01-1.47) | 1.23  (1.02-1.48) | 1.15  (0.96-1.39) |
| 61-120 | 1.52  (1.28-1.79) | 1.54  (1.30-1.83) | 1.39  (1.17-1.65) |
| >120 | 2.31  (1.94-2.75) | 2.38  (2.00-2.84) | 2.00  (1.67-2.40) |
| *P_trend_* | <.001 | <.001 | <.001 |
| Presleep mobile phone use time(increase by 10 minutes per day) | 1.04  (1.03-1.05) | 1.04  (1.03-1.05) | 1.04  (1.03-1.04) |
| Postwake mobile phone use time(minutes per day) |  |  |  |
| 0-1 | reference | reference | reference |
| 2-10 | 1.26  (1.06-1.51) | 1.24  (1.04-1.49) | 1.17  (0.98-1.40) |
| 11-30 | 1.21  (1.02-1.45) | 1.21  (1.01-1.44) | 1.10  (0.92-1.31) |
| ＞30 | 1.52  (1.28-1.79) | 1.52  (1.28-1.80) | 1.33  (1.12-1.68) |
| *P_trend_* | <.001 | <.001 | .003 |
| Postwake mobile phone use time(increase by 10 minutes per day) | 1.02  (1.01-1.03) | 1.02  (1.02-1.03) | 1.02  (1.01-1.03) |

^a^model 1: unadjusted;

^b^model 2: adjustment for sex, grade, ethnicity, registered permanent residence, sibship, maternal educational attainment, and paternal educational attainment;

^c^model 3: adjustment for sex, grade, ethnicity, registered permanent residence, sibship, maternal educational attainment, paternal educational attainment, smoking, drinking, unhealthy diet, and less physical activity.
